# Integrated safety profile of selinexor in multiple myeloma: experience from 437 patients enrolled in clinical trials

**DOI:** 10.1038/s41375-020-0756-6

**Published:** 2020-02-24

**Authors:** Maria Gavriatopoulou, Ajai Chari, Christine Chen, Nizar Bahlis, Dan T. Vogl, Andrzej Jakubowiak, David Dingli, Robert F. Cornell, Craig C. Hofmeister, David Siegel, Jesus G. Berdeja, Donna Reece, Darrell White, Suzanne Lentzsch, Cristina Gasparetto, Carol Ann Huff, Sundar Jagannath, Rachid Baz, Ajay K. Nooka, Joshua Richter, Rafat Abonour, Terri L. Parker, Andrew J. Yee, Philippe Moreau, Sagar Lonial, Sascha Tuchman, Katja C. Weisel, Mohamad Mohty, Sylvain Choquet, T. J. Unger, Kai Li, Yi Chai, Lingling Li, Jatin Shah, Sharon Shacham, Michael G. Kauffman, Meletios Athanasios Dimopoulos

**Affiliations:** 1 Alexandra Hospital, School of Medicine, National and Kapodistrian University of Athens, Athens, Greece; 2 Tisch Cancer Institute, Icahn School of Medicine at Mount Sinai, New York, NY USA; 3 Division of Medical Oncology & Hematology, Princess Margaret Cancer Centre, Toronto, ON Canada; 4 Charbonneau Cancer Research Institute, Calgary, AB Canada; 5 Abramson Cancer Center, Perelman School of Medicine, University of Pennsylvania, Philadelphia, PA USA; 6 University of Chicago Medical Center, Chicago, IL USA; 7 Mayo Clinic, Rochester, MN USA; 8 Vanderbilt University Medical Center, Nashville, TN USA; 9 Department of Internal Medicine, Division of Hematology, The Ohio State University, Columbus, OH USA; 10 Department of Hematology, John Theurer Cancer Center, Hackensack, NJ USA; 11 Sarah Cannon Research Institute, Nashville, TN USA; 12 QEII Health Sciences Center, Dalhousie University, Halifax, NS Canada; 13 Department of Medicine, Division of Hematology/Oncology, Columbia University, New York, NY USA; 14 Duke University Cancer Center, Durham, NC USA; 15 Johns Hopkins University, Baltimore, MD USA; 16 Department of Malignant Hematology, H. Lee Moffitt Cancer Center and Research Institute, Tampa, FL USA; 17 Winship Cancer Institute, Emory University, Atlanta, GA USA; 18 Indiana University Cancer Center, Indianapolis, IN USA; 19 Yale School of Medicine, New Haven, CT USA; 20 Massachusetts General Hospital Cancer Center, Boston, MA USA; 21 University of Nantes, Nantes, France; 22 Lineberger Comprehensive Cancer Center, University of North Carolina, Chapel Hill, NC USA; 23 University Medical Center of Hamburg-Eppendorf, Hamburg, Germany; 24 Hôpital Saint Antoine, Paris, France; 25 La Pitié–Salpêtrière Hospital, Paris, France; 26 Karyopharm Therapeutics, Newton, MA USA

**Keywords:** Myeloma, Adverse effects

## Abstract

Selinexor is an oral, small molecule inhibitor of the nuclear export protein exportin 1 with demonstrated activity in hematologic and solid malignancies. Side effects associated with selinexor include nausea, vomiting, fatigue, diarrhea, decreased appetite, weight loss, thrombocytopenia, neutropenia, and hyponatremia. We reviewed 437 patients with multiple myeloma treated with selinexor and assessed the kinetics of adverse events and impact of supportive care measures. Selinexor reduced both platelets and neutrophils over the first cycle of treatment and reached a nadir between 28 and 42 days. Platelet transfusions and thrombopoietin receptor agonists were effective at treating thrombocytopenia, and granulocyte colony stimulating factors were effective at resolving neutropenia. The onset of gastrointestinal side effects (nausea, vomiting, and diarrhea) was most common during the first 1–2 weeks of treatment. Nausea could be mitigated with 5-HT3 antagonists and either neurokinin 1 receptor antagonists, olanzapine, or cannbainoids. Loperamide and bismuth subsalicylate ameliorated diarrhea. The primary constitutional side effects of fatigue and decreased appetite could be managed with methylphenidate, megestrol, cannabinoids or olanzapine, respectively. Hyponatremia was highly responsive to sodium replacement. Selinexor has well-established adverse effects that mainly occur within the first 8 weeks of treatment, are reversible, and respond to supportive care.

## Introduction

Multiple myeloma (MM) is characterized by the aberrant, clonal expansion of malignant plasma cells, resulting in hypercalcemia, renal impairment, anemia, bone lesions, and susceptibility to infections. It is estimated that 138,500 people are living with myeloma worldwide and over 98,400 will succumb to the disease this year [1]. Though MM remains largely incurable, advances in autologous hematopoietic cell transplantation, as well as novel therapies including proteasome inhibitors, immunomodulatory agents, monoclonal antibodies, and histone deacetylase inhibitors, have improved response rates and survival of patients over the past two decades [2–7]. These drugs have novel mechanisms of action compared with classical chemotherapies, and therefore unique toxicity profiles that require experience and supportive care strategies to manage them effectively. The toxicities of current antimyeloma therapies can be broadly classified as hematologic, gastrointestinal, neurological, cardiac, hepatic, immunologic, and teratogenic [8]. Knowledge of the optimal dose levels, routes of administration, and refinement of chemical structures have improved the effectiveness of these agents and treatment experience for patients over time [9–12].

Selinexor is an oral selective inhibitor of exportin 1 (XPO1) that has recently been approved by the US Food and Drug Administration for the treatment of patients with refractory MM [13]. In most cancer cells, including MM, XPO1 is overexpressed and associated with poor prognosis [14, 15]. Selinexor reversibly binds to a critical cysteine residue (cys-528) in the nuclear export sequence-binding groove of XPO1 and prevents the transport of over 200 cargo proteins from the nucleus to the cytoplasm [16]. Inhibition of XPO1 retains tumor suppressor proteins in the nucleus leading to their activation, and prevents the nuclear export of mRNAs coding for oncoproteins, such as Myc, Bcl-6, and cyclin D1 [17]. These effects are lethal to cancer cells at concentrations that are not toxic to normal cells [18].

To date, selinexor has been administered to over 3000 patients with hematologic and solid cancers in clinical trials as a single agent or in combination with other therapies. The side effect profile of selinexor is well-established and predictable. The most common adverse events (AEs) are gastrointestinal (nausea, vomiting, and diarrhea), constitutional (fatigue, decreased appetite), hematologic (thrombocytopenia and neutropenia), and biochemical (hyponatremia) [19, 20]. These AEs are largely dose and schedule dependent, reversible, and there is no evidence of major organ or cumulative toxicities after long-term treatment. AEs can be prevented or mitigated with prophylactic measures, and vigilant monitoring and management. Early incorporation of supportive care is key to address these side effects and maintain the patient’s quality of life [19, 20].

Here, we describe a pooled analysis of patients with MM treated with selinexor, alone or in combinations, across four clinical trials. The objective of this analysis was to explore the effectiveness of supportive care agents and better understand the kinetics of the AE onset and resolution with and without intervention.

## Methods

### Integrated analysis

This was a retrospective, pooled analysis of 437 patients with MM from the phase 1 (NCT01607892) (*N* = 81), STORM (NCT02336815) (*N* = 202), STOMP (NCT02343042) (*N* = 117), and BOSTON (NCT03110562) (*N* = 37) trials. Inclusion and exclusion criteria for each trial are listed in Supplementary Table 1. The study protocols were approved by the institutional review board or an independent ethics committee at each participating center and were in accordance with the Declaration of Helsinki and the International Conference on Harmonization-Good Clinical Practice. All patients provided written informed consent prior to enrollment. Treatment-emergent adverse events (TEAE) were defined as any AE that developed, worsened, or became serious during the treatment period (time from first dose of selinexor (C1D1) to last dose of selinexor plus an additional 30 days of follow-up) regardless of causality. AEs were graded by the treating physician according to the Common Terminology Criteria for Adverse Events (CTCAE) version 4.03. An AE was considered resolved/resolving if there was an improvement in severity to a lower grade or recovery of an AE to normal without sequelae.

## Results

### Demographics

Baseline characteristics of all patients included in the analysis are outlined in Table 1. Patients had a median age of 64 years (range: 34–85), had a median time since initial MM diagnosis of 5 years (range: <1–35), and were heavily pretreated (69% ≥5 prior lines of therapy). Of note, patients had a median of 13 (range: 1–62) distinct abnormalities in baseline clinical and laboratory tests at C1D1 of a selinexor trial with 43% having thrombocytopenia, 38% fatigue/asthenia, 27% neutropenia, and 14% diarrhea.

**Table 1:** Characteristics of patients at screening.

Characteristic	All patients
	*N* = 437
Age (years)	
Median (range)	64 (34–85)
≤65	229 (52%)
65–74	160 (37%)
≥75	48 (11%)
Sex	
Male	236 (54%)
Female	201 (46%)
Race	
White	341 (78%)
Black or African American	55 (13%)
Asian	10 (2%)
Other/unknown	31 (7%)
ECOG performance status at screening	
0	108 (25%)
1	280 (64%)
2	38 (9%)
3	1 (<1%)
Unknown	10 (2%)
ISS disease stage at initial diagnosis	
I	95 (22%)
II	87 (20%)
III	127 (29%)
IV	2 (<1%)
Unknown/missing	126 (29%)
Median time since initial diagnosis of multiple myeloma	
Years (range)	5 (<1–35)
Number of prior therapeutic regimens	6 (0–18)
0	2 (<1%)
1	42 (10%)
2	27 (6%)
3	26 (6%)
4	37 (8%)
5	56 (13%)
≥6	247 (57%)
Selinexor starting dose and treatment regimen	
Selinexor (80 mg) + dexamethasone (20 mg)^a^	214 (49%)
* KCP-330-001*	12 (6%)^b^
* KCP-330-012 Part A*	79 (37%)^b^
* KCP-330-012 Part B*	123 (57%)^b^
Selinexor (< or >80 mg) ± dexamethasone (20 mg)	70 (16%)
Selinexor/bortezomib/dexamethasone^c^	78 (18%)
Selinexor/lenalidomide/dexamethasone^d^	24 (5%)
Selinexor/pomalidomide/dexamethasone^e^	33 (8%)
Selinexor/daratumumab/dexamethasone^f^	18 (4%)
Abnormalities in baseline clinical and laboratory tests	
Median (range)	13 (1–62)
Nausea at screening	
None	386 (88%)
Grade 1	31 (7%)
Grade 2	4 (<1%)
Ongoing with unknown grade	16 (4%)
Diarrhea at screening	
None	378 (86%)
Grade 1	36 (8%)
Grade 2	6 (1%)
Ongoing with unknown grade	17 (4%)
Fatigue or asthenia at screening	
None	273 (62%)
Grade 1	95 (22%)
Grade 2	18 (4%)
Ongoing with unknown grade	51 (12%)
Decreased appetite at screening	
None	411 (94%)
Grade 1	16 (4%)
Grade 2	4 (<1%)
Ongoing with unknown grade	6 (1%)
Platelet count at screening	
Above lower limit of normal	249 (57%)
<150,000–75,000/mm^3^	117 (27%)
<75,000–50,000/mm^3^	39 (9%)
<50,000–25,000/mm^3^	24 (6%)
<25,000/mm^3^	6 (1%)
Neutrophil count at screening	
Above lower limit of normal	315 (72%)
<2000–1500/mm^3^	64 (15%)
<1500–1000/mm^3^	40 (9%)
<1000–500/mm^3^	10 (2%)
<500/mm^3^	2 (<1%)
Hemoglobin at screening	
Above lower limit of normal	46 (11%)
<Lower limit of normal–10.0 g/dL	182 (42%)
<10.0–8.0 g/dL	174 (40%)
<8.0 g/dL	34 (8%)
Plasma sodium at screening	
Above lower limit of normal	411 (94%)
Grade 1	18 (4%)
Grade 2	1 (<1%)
Grade 3	4 (<1%)
Ongoing with unknown grade	3 (<1%)
Concomitant medications at screening	
Median (range)	7 (0–21)

*ECOG* Eastern Cooperative Oncology Group, *ISS* International Staging System.

^a^Percentage of *N* = 214.

^b^Selinexor (80 mg twice-weekly)/dexamethasone (20 mg twice-weekly).

^c^Selinexor (60 or 80 mg twice-weekly, or 80 or 100 mg once-weekly)/bortezomib (1.3 mg/m^2^ once-weekly or twice-weekly/dexamethasone (20 mg twice-weekly or 40 mg once-weekly).

^d^Selinexor (60 or 80 mg twice-weekly, or 80 or 100 mg once-weekly)/lenalidomide (25 mg once-daily)/dexamethasone (20 mg twice-weekly or 40 mg once-weekly).

^e^Selinexor (60 or 80 mg twice-weekly, or 60, 80 or 100 mg once-weekly)/pomalidomide (2, 3, or 4 mg once-daily)/dexamethasone (20 mg twice-weekly or 40 mg once-weekly).

^f^Selinexor (60 mg twice-weekly or 100 mg once-weekly/daratumumab (intravenous, 16 mg/kg once-weekly)/dexamethasone (20 mg twice-weekly or 40 mg once-weekly).

Sixty-seven percent of the patients received selinexor on a twice-weekly schedule, 27% on a once-weekly schedule, and 6% on another schedule (primarily during the dose-escalation phase of trials). The average starting dose received was 61–80 mg/dose for 51% of patients, 81–100 mg/dose for 25% of patients, >100 mg/dose for 3% of patients, and <60 mg/dose for 21% of patients. The median weekly dose for all patients was 100 mg (range: 7–248 mg).

### Thrombocytopenia

Decreased platelet count in patients with MM was the most common hematologic AE associated with selinexor treatment. Among the 437 patients in this analysis, 66% of patients developed thrombocytopenia of any grade (<150,000 platelets/mm^3^), 22% developed grade 3 thrombocytopenia (<50,000–25,000 platelets/mm^3^), and 32% developed grade 4 thrombocytopenia (<25,000 platelets/mm^3^) while on study (Supplementary Table 2).

**Fig. 1:**
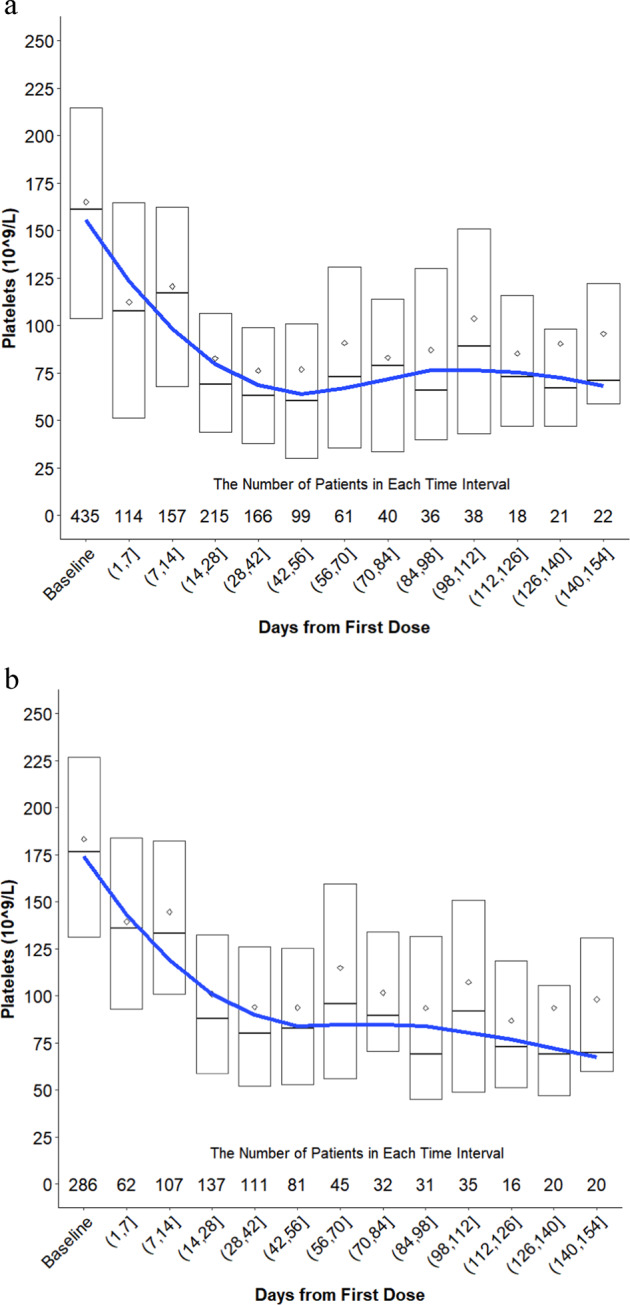
Platelet change in patients during selinexor treatment. **a** Change in platelets from baseline (cycle 1 day 1 of treatment with selinexor or a selinexor-containing regimen) through day 154 for all patients with MM. Patients were included in the graph up until the point in time when they received an intervention for thrombocytopenia (platelet transfusion, TPO receptor agonist, dose reduction, and dose interruption) and were then subsequently removed from the analysis. **b** Change in platelets for all patients who did not receive a platelet transfusion or TPO receptor agonist while on a selinexor trial. Patients who received a dose reduction or interruption were included in the analysis up until the point in time where they had an intervention and were subsequently removed from the analysis.

Platelet decrease was evident within the first 7 days of treatment and reached a nadir between 28 and 42 days in the absence of intervention (e.g., platelet transfusion, thrombopoietin (TPO) receptor agonist (TPO-RA), and/or dose reduction/interruption) Fig. 1a, b. Patients had a median baseline platelet count of 161,000 (quartile Q1 104,000, Q3 214,000) and a median decrease of 85,000 platelets/mm^3^ (Q1 45,000, Q3 141,000) from the start of selinexor through days 28–42. Importantly, for patients who continued therapy, there was no subsequent decrease in platelets from days 42 to 154 of treatment. The median time to onset of the first recorded thrombocytopenia was 22 days (Q1 12, Q3 29 days) and median time to onset of grade ≥3 thrombocytopenia was 29 days (Q1 16, Q3 40 days). Severe bleeding (grade ≥3) with corresponding grade ≥3 thrombocytopenia was uncommon (<3%). For patients who did not have an intervention for thrombocytopenia, 68 (44%) of the AEs resolved or were resolving with the median duration of the AE being 52 days (Q1 24, Q3 134).

There were four strategies used to manage thrombocytopenia: platelet transfusions, TPO-RAs, dose reductions, and dose interruptions (Supplementary Fig. 1a–d). Platelet transfusions were primarily instituted for grade 4 thrombocytopenia (median 23,000 platelets/mm^3^ at time of platelet transfusion; Q1 17 000, Q3 42 000). In total, 119 (27%) patients had a platelet transfusion while on study. Eighty two (77%) of the 107 patients that received a platelet transfusion and had a subsequent assessment showed resolution of thrombocytopenia in a median time of 8 days (Q1 3, Q3 16 days).

In preclinical models, TPO-RAs are effective at stimulating platelet production [21–23]. The clinical trials permitted use of the TPO-RAs romiplostim or eltrombopag to manage selinexor-induced thrombocytopenia, and 48 (11%) patients received one of these agents. The median starting doses of eltrombopag and romiplostim were 50 mg (Q1 50, Q3 50 mg) and 1 μg/kg (Q1 1, Q3 2 μg/kg), respectively. The dose of romiplostim was incrementally increased each subsequent week up to 10 μg/kg if platelet counts were not restored. In general, higher doses of romiplostim or eltrombopag (≥5 µg/kg and ≥100 mg, respectively) were associated with a greater increase in platelet counts than lower doses. In addition, TPO-RAs were more effective with a once-weekly dosing schedule of selinexor compared with twice-weekly. The median time to first use of any TPO-RA was 36 days from baseline (Q1 29, Q3 45 days). The first evidence of thrombopoiesis for most patients occurred between 14 and 28 days and platelets steadily increased. Of the 48 patients who had a TEAE of thrombocytopenia and received a TPO-RA, 67% had their AE resolved or resolving in a median time of 14 days (Q1 7, Q3 23 days).

Overall, both dose reduction and interruption were effective at mitigating thrombocytopenia; however, platelet loss persisted for an additional 7–14 days after dose reduction or interruption (~22,000 platelets/mm^3^ to nadir). The median time to first dose reduction and interruption for thrombocytopenia occurred at 36 days (range: 8–722 days) and 32 days (range: 4–1021 days), respectively. Dose reductions for thrombocytopenia occurred in 141 (32%) patients with the most common dose and schedule change from selinexor 80 mg twice-weekly to 100 mg once-weekly.

### Neutropenia

Decreased neutrophil count occurred in 163 (37%) patients (<2000 neutrophils/mm^3^) with 85 (20%) patients having grade 3 neutropenia (<1000–500 neutrophils/mm^3^) and 38 (9%) having grade 4 neutropenia (<500 neutrophils/mm^3^) while on study (Supplementary Table 2). Febrile neutropenia occurred in 19 (4%) of the 437 patients in this analysis. Severe (grade ≥3) infections in the context of grade ≥3 neutropenia occurred in 23 patients (19%) and included *Clostridium difficile* infection (1 patient), bacteremia (2 patients), upper respiratory tract infection (2 patients), influenza (3 patients), sepsis (3 patients), pneumonia, or lung infection (12 patients).

**Fig. 2:**
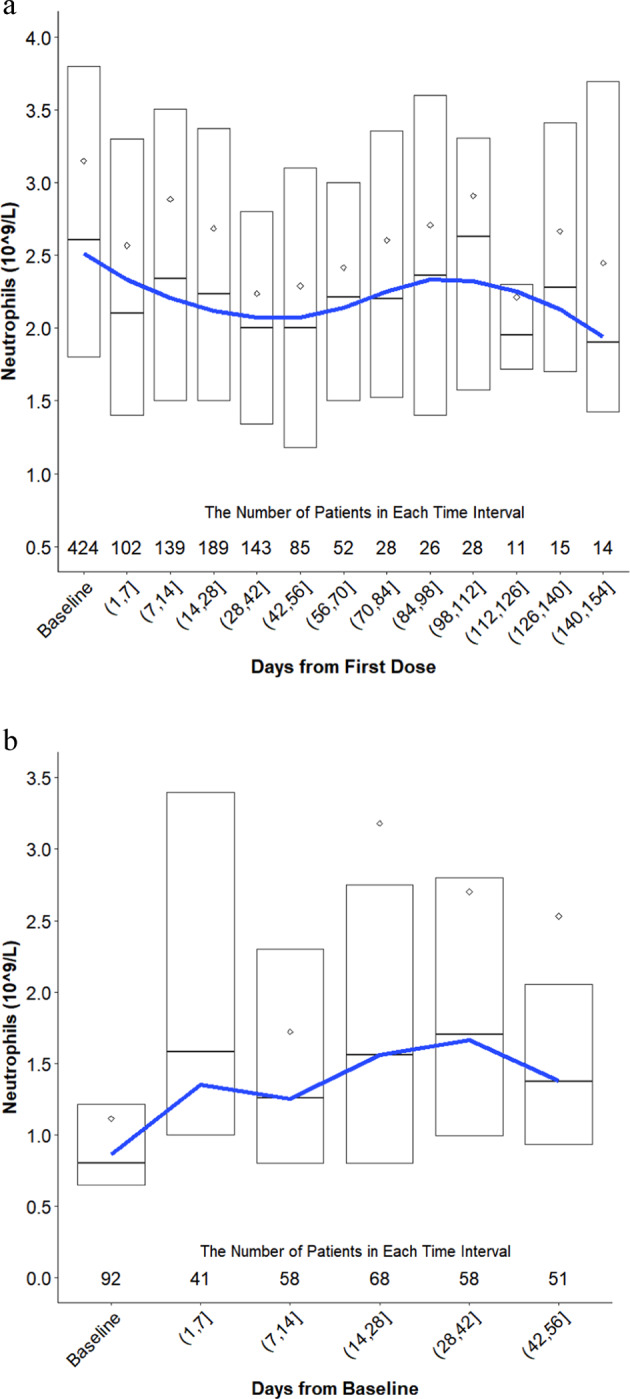
Neutrophil change in patients during selinexor treatment and after a G-CSF. **a** Change in neutrophils from baseline (C1D1 of treatment with selinexor or a selinexor-containing regimen) through day 154 for all patients with MM. Patients were included in the graph up until the point in time when they received an intervention for neutropenia (filgrastim/pegfilgrastim, dose reduction, and dose interruption) and were then subsequently removed from the analysis. **b** Change in neutrophils for all patients who received filgrastim or pegfilgrastim.

Neutropenia began within the first 7 days of treatment and reached a nadir between 28 and 42 days in the absence of intervention (granulocyte colony stimulating factor (G-CSF) or dose reduction/interruption) (Fig. 2a). The median time to onset of any grade of neutropenia was 21 days (Q1 8, Q3 43 days) and was 26 days (Q1 15, Q3 50 days) for grade ≥3. The median duration of neutropenia among all patients, regardless of intervention, was 10 days (Q1 7, Q3 18). Patients had a median decrease of 530 neutrophils/mm^3^ (Q1 0, Q3 1700 neutrophils/mm^3^) from the start of selinexor through days 28–42. There was no substantial decrease in neutrophil count between days 42 and 154 of treatment.

The G-CSF agents filgrastim and pegfilgrastim were used in 92 (75%) patients with grade ≥3 neutropenia and were most commonly administered as a single injection (median 1 dose; Q1 1, Q3 3 doses) at 300 µg or 6 mg subcutaneously, respectively. Both filgrastim and pegfilgrastim were effective at rapidly (within 7 days) and durably raising neutrophil counts (Fig. 2b). These supportive care agents were instituted at a median of 800 neutrophils/mm^3^ and increased neutrophil levels to 1580 neutrophils/mm^3^ (Q1 1000, Q3 3400) within the first week. Neutrophil levels continued to improve over the subsequent weeks. Neutropenia resolved or was resolving in 90% of patients that received filgrastim or pegfilgrastim versus 64% of patients that did not. Patients who received a G-CSF had a median time to resolution of neutropenia of 8 days (Q1 5, Q3 15).

### Nausea/vomiting

The most common AE observed with selinexor treatment is nausea, which occurred in 68% of patients with grade 3 nausea in 6% of patients; vomiting occurred in 37% of patients. The median time to onset of any grade nausea/vomiting (N/V) was 3 days (Q1 2, Q3 8) and median time to onset of grade 3 N/V was 16 days (Q1 3, Q3 31 days). For all patients, the onset of N/V was highest within the first cycle of treatment (70%) and dropped substantially for each subsequent cycle. Fifty-eight percent of patients with N/V had corresponding fatigue, 51% decreased appetite, 34% weight loss, 27% hyponatremia, and 17% dehydration.

The average duration of N/V was 22 days (Q1 6, Q3 51) for patients who did not receive supportive care within 5 days of onset and was 13 days (Q1 5, Q3 34 days) for patients who did receive such care. A full summary of the supportive care agents that were used to manage N/V can be found in Table 2. Most patients received a mandatory 5-HT3 antagonist on the day of and day after dosing. Additional agents improved the frequency of resolution and substantially reduced the median duration of N/V. In particular, neurokinin 1 (NK1) RAs (rolapitant [180 mg], aprepitant [80 mg], or fosaprepitant [150 mg] prior to study drug administration and as needed), benzodiazepenes (lorazepam 0.5–1 mg daily as needed), and cannabinoid-RAs (dronabinol 2.5–5 mg twice-daily as needed) were effective at resolving N/V and/or substantially reducing the duration of this AE.

**Table 2:** Supportive care agents by drug class administered within 5 days of onset of nausea/vomiting and outcomes of patients after intervention.

	No supportive care	Serotonin receptor antagonist	Dopamine receptor (D2/D3) antagonist	NK1 antagonist	Benzodiazepine	GR receptor agonist	Cannabinoid receptor agonist	Histamine (H1) receptor agonist	Olanzapine
*N* (%) of patients with any grade nausea/vomiting	217 (69%)	118 (38%)	54 (17%)	25 (8%)	26 (8%)	39 (12%)	10 (3%)	18 (6%)	13 (4%)
Grade 1 *N* (%)	97 (45%)	61 (52%)	29 (54%)	9 (36%)	10 (39%)	16 (41%)	1 (10%)	6 (33%)	5 (39%)
Grade 2 *N* (%)	104 (48%)	47 (40%)	19 (35%)	10 (40%)	13 (50%)	21 (54%)	7 (70%)	10 (56%)	7 (54%)
Grade 3 *N* (%)	16 (7%)	10 (9%)	6 (11%)	6 (24%)	3 (12%)	2 (5%)	2 (20%)	2 (11%)	1 (8%)
AEs resolved/resolving *N* (%)	122 (56%)	70 (59%)	35 (65%)	18 (72%)	18 (69%)	14 (36%)	9 (90%)	10 (56%)	10 (77%)
AEs Not resolved/resolving *N* (%)	95 (44%)	48 (41%)	19 (35%)	7 (28%)	8 (31%)	25 (64%)	1 (10%)	8 (44%)	3 (23%)
Median duration of event for resolved/resolving nausea/vomiting (days; Q1, Q3)	22 (6, 51)	8 (4, 29)	7 (3, 29)	9 (2, 53)	6 (3, 72)	13 (1, 31)	12 (8, 20)	11 (3, 23)	15 (5, 45)

Incidence and severity of nausea/vomiting at the time of supportive care with outcomes of patients who had intervention within 5 days of AE onset. Supportive care agents per drug class: serotonin (5-HT3) receptor agonist (granisetron, mirtazapine, ondansetron, palonosetron), dopamine (D2/D3) receptor antagonist (alizapride, domperidone, haloperidol, metoclorpramide, prochloroperazine, thiethylperazine maleate, trimethobenzamide), neurokinin 1 (NK1) receptor antagonists (rolapitant, aprepitant, fosaprepitant), benzodiazepine (clonazepam, diazepam, lorazepam), glucocorticoid receptor (GR) agonist (dexamethasone, prednisolone, prednisone), cannabinoid receptor agonist (dronabinol, nabilone, and cannabis sativa), and histamine (H1) receptor agonist (cyclizine, dimenhydrinate, diphenhydramine, meclizine, promethazine).

Consistent with a previous report, prophylactic olanzapine (2.5–5 mg orally in the evening) or megestrol (400 mg daily) for N/V (and decreased appetite discussed below) were used in 39 (9%) patients [24]. Incidence and severity of N/V were numerically lower in patients who received prophylactic olanzapine and megestrol (any grade, 56%; grade 3, 3%), compared with patients who did not (any grade, 70%; grade 3, 7%).

### Diarrhea

There were 177 (41%) patients who reported diarrhea while taking selinexor with a median duration of 4 days (Q1 3, Q3 15 days). Twenty-two (5%) of the patients had grade 3 diarrhea with no grade ≥4. The median time to onset for the first event of any grade diarrhea was 13 days (Q1 5, Q3 36 days). Seventy-two percent of reported cases of diarrhea were resolved or resolving in a median time of 7 days (Q1 3, Q3 24 days). In general, diarrhea was associated with fatigue (45%), decreased appetite (40%), decreased body weight (24%), and hyponatremia (21%). Grade 3 diarrhea was most commonly associated with grade ≥3 hyponatremia (18%) and fatigue (9%). Diarrhea resolved or was resolving in 87% of patients who received either loperamide or bismuth subsalicylate.

### Fatigue

Fatigue is a common AE among patients with heavily pretreated MM and was one of the most frequently observed TEAEs in selinexor trials. Importantly, 38% of the 437 patients analyzed in this study had ongoing fatigue during the screening period prior to selinexor treatment. Overall, 63% of patients reported any grade of fatigue while on a selinexor trial, with a median time-to-onset of 7 day (Q1 3, Q3 20 days); 16% had grade 3 fatigue with a median time to onset of 22 days (Q1 8, Q3 50). In the majority (70%) of patients, fatigue did not resolve, but for those in whom fatigue resolved or was resolving, the median duration of this AE was 29 days (Q1 11, Q3 67 days). Fatigue was most commonly associated with decreased appetite (54%), anemia (48%), decreased body weight (34%), and dehydration (15%).

Methylphenidate was used either prophylactically or on study in 12 patients treated with selinexor. Of these 12 patients, three were treated upon emergence of fatigue with daily doses of methylphenidate ≤5 mg, and none saw a resolution of or improvement in fatigue (Supplementary Table 3). The remaining nine patients were treated at doses ≥10 mg of methylphenidate. Three patients were treated prophylactically, and none reported fatigue while on study. The other six received methylphenidate as needed, with five of the six patients having a resolution of their fatigue. The median time to resolution of fatigue with methylphenidate was 6 days (Q1 4, Q3 8 days).

For fatigue that was attributed to insomnia, mirtazapine or olanzapine were used in 13 patients. Fatigue resolved or was resolving in 8 (62%) of these patients with a median duration of 10 days (Q1 6, Q3 42 days).

### Decreased appetite/weight loss

Decreased appetite occurred in 53% of patients, with grade 3 in 7%. The median time to onset of decreased appetite was 8 days (Q1 3, Q3 22 days), and the median time to onset for grade ≥3 was 35 days (Q1 24, Q3 44 days). The primary AEs that were associated with decreased appetite were fatigue (66%), nausea (59%), weight loss (47%), diarrhea (41%), vomiting (33%), dehydration (17%), and dysgeusia (17%). In 56% of patients, decreased appetite did not resolve without intervention. For the 46% with decreased appetite that resolved or resolving in the absence of intervention, the median duration was 36 days (Q1 15, Q3 109 days).

The primary supportive care measures to manage decreased appetite and accompanied weight loss were the use of megestrol (10%), cannabinoids (6%) (cannabis sativa, dronabinol), or olanzapine or mirtazapine (5%) (Table 3). Typical doses were 400 mg daily megestrol acetate, 2.5 mg twice-daily dronabinol, or 5 mg nightly olanzapine. For patients who received supportive care within 5 days of onset of decreased appetite, the frequency of AE resolution was 64% with megestrol, 77% with cannabinoids, and 50% with olanzapine or mirtazapine. The average duration of decreased appetite was 21 days (Q1 8, Q3 29 days) with megestrol, 41 days (Q1 19, Q3 54 days) with cannabinoids, and 8 days (Q1 6, Q3 25 days) with olanzapine or mirtazapine. Of the 39 patients who received prophylactic olanzapine and megestrol, 31% had decreased appetite versus 50% who did not receive such prophylaxis.

**Table 3:** Supportive care agents by drug class administered within 5 days of onset of decreased appetite and outcomes of patients after intervention.

	No supportive care	Megestrol acetate	Cannabinoid receptor agonist	Glucocorticoid receptor agonist	Mirtazapine or olanzapine
*N* (%) of patients with any grade decreased appetite	197 (53%)	22 (10%)	13 (6%)	26 (11%)	12 (5%)
Grade 1 *N* (%)	87 (44%)	6 (27%)	3 (23%)	10 (39%)	4 (33%)
Grade 2 *N* (%)	96 (49%)	15 (68%)	10 (77%)	11 (42%)	7 (58%)
Grade 3 *N* (%)	14 (7%)	1 (5%)	–	5 (19%)	1 (8%)
AEs resolved/resolving *N* (%)	87 (44%)	14 (64%)	10 (77%)	8 (31%)	6 (50%)
AEs not resolved/resolving *N* (%)	110 (56%)	8 (36%)	3 (23%)	18 (69%)	6 (50%)
Median duration of event for resolved decreased appetite (days; Q1, Q3)	36 (15, 109)	21 (8, 29)	41 (19, 54)	14 (3, 33)	8 (6, 25)

Incidence and severity of decreased appetite at the time of supportive care with outcomes of patients who had intervention within 5 days of AE onset. Supportive care agents per drug class: cannabinoid receptor agonist (dronabinol, nabilone, cannabis sativa, glucocorticoid receptor (GR) agonist (dexamethasone, prednisolone, prednisone).

### Hyponatremia

Hyponatremia (sodium <135 mmol/L) was observed in 138 (32%) of the 437 patients while on study and 83 (19%) patients had grade ≥3 hyponatremia (120 to <130 mmol/L); there is no grade 2 hyponatremia in CTCAE v4.03. The mechanism behind treatment-emergent hyponatremia is not completely understood and the etiology is multifactorial. Importantly, a majority of grade ≥3 hyponatremia was asymptomatic, with <5% of the cases associated with grade ≥3 dizziness, confusion, altered mental status, or delirium.

The median time to onset of any grade of hyponatremia was 8 days (Q1 5, Q3 18 days) with the median time to onset of grade ≥3 hyponatremia being 10 days (Q1 6, Q3 24 days). The primary supportive care measure for managing hyponatremia was sodium chloride tablets. Of the 60 patients who received this supportive care, 83% had resolution of hyponatremia versus 65% that did not receive sodium chloride. Furthermore, the median time to resolution of hyponatremia was 7 days (Q1 3, Q3 13) for patients who received sodium chloride and 12 days (Q1 6, Q3 20 days) for patients who did not.

## Discussion

This is the first paper to describe a set of pooled AE data from patients with MM treated with selinexor as a single agent or in combination. We show the kinetics of the most common selinexor TEAEs and provide practice-based prophylactic and supportive care strategies to address them (Table 4). The most common side effects were reversible and consistent with previous reports and included thrombocytopenia, neutropenia, nausea, vomiting, diarrhea, fatigue, decreased appetite, and hyponatremia [19, 25–27]. Most patients who were treated with selinexor required concomitant prophylactic and on study supportive care, dose modifications, and vigilant monitoring to prevent or avoid worsening of AEs. Clinical sequellae of cytopenias and low sodium were not commonly observed, and major organ (cardiac, hepatic, renal, neural) or cumulative toxicities were not typical.

**Table 4:** Summary of recommended supportive care guidelines and patient management with selinexor treatment.

Patient management	Weekly monitoring of blood counts, serum sodium, and body weight for the first 8 weeks and as needed thereafter.
Prophylaxis for Nausea and/or Vomiting^a^	All patients should receive a 5-HT3 receptor antagonist (e.g., ondansetron, 8 mg, PO, 30 min prior to first dose of selinexor, with subsequent 8 mg dose 8 h after the first dose of selinexor; then administered 8 mg twice-daily (every 12 h with coverage for 24 h after the last dose of selinexor); continued use should be evaluated after the first 8 weeks. Patients at high-risk for nausea and/or vomiting, or decreased appetite should receive an additional agent such as olanzapine (5–10 mg, PO, once-daily in the evening) or NK1 receptor antagonist (e.g., rolapitant per label). If using aprepritant or fosapreptiant the dose of dexamethasone may need to be reduced. Olanzapine or NK1 receptor antagonists may be reduced or stopped after the first 8 weeks if the patient is tolerating selinexor treatment.
Diarrhea	Loperamide (4 mg for the first dose, and 2 mg thereafter, PO, as needed) until diarrhea resolves.
Hyponatremia	Hydration status and serum sodium should be monitored, and saline/salt tablets employed as needed.
Thrombocytopenia^b^	TPO receptor agonist for platelet counts below 25,000/mm^3^ (romiplostim, 5–10 mcg/kg, IV, once-weekly; or eltrombopag, 50 mg, PO, daily, until platelet counts recover to ≥50,000/mm^3^).
Neutropenia^c^	G-CSF for ANC below 500/mm^3^ (filgrastim, SC or IV, 5 mcg/kg), daily until neutrophil count resolves to >1000/mm^3^; or pegfilgrastim, SC, 6 mg, weekly until neutrophil count resolves to >1000/mm^3^).
Fatigue^d^	Establish causative relationship between treatment regimen and fatigue (consider onset, pattern, duration, change over time, as well as the patient’s disease status and self-assessment of causes of fatigue). For grade 2 or 3 fatigue, methylphenidate (10 mg, PO, daily) as needed.

^a^Follow NCCN and/or European Society for Medical Oncology (ESMO) guidelines for moderate emetogenic chemotherapies (MECs) and treat for both acute and delayed emesis.

^b^Recommendation is off-label use of romiplostim and eltrombopag.

^c^Follow 2019 NCCN guidelines: hematopoietic growth factors.

^d^Consider 2019 NCCN guidelines for the treatment of cancer related fatigue.

The primary hematological AEs with selinexor treatment are thrombocytopenia and, to a lesser extent, neutropenia. Associated bleeding or febrile neutropenia were uncommon. Preclinical studies have demonstrated that selinexor prevents hematopoietic stem and progenitor cell maturation into megakaryocytes through inhibition of TPO signaling and accumulation of phosphorylated STAT3 in the nucleus, rather than direct cytotoxic effects on hematopoietic stem cells, megakaryocytes, or platelets [21]. When considering the patient population in this analysis, it is important to note that in addition to substantial marrow involvement by their myeloma, nearly all of the patients had received previous myelosuppressive treatments, and 43% and 28% of the patients had preexisting thrombocytopenia and neutropenia, respectively. Previous reports showed that platelet counts at baseline were predictive of developing high-grade thrombocytopenia, with nearly all patients with platelet counts <75,000/mm^3^ at baseline having grade ≥3 thrombocytopenia while on study [26]. Physicians should consider weekly monitoring of blood counts, particularly in patients with low baseline counts. The data show that platelet transfusions were effective at rapidly boosting platelet levels for patients with severe thrombocytopenia, and that romiplostim or eltrombopag, albeit off-label use of these drugs, could be used to increase platelet counts over 2–3 weeks, often while continuing selinexor treatment. G-CSF was highly effective at rapidly normalizing low neutrophil counts.

Gastrointestinal AEs, in particular N/V, are the most common nonhematological side effects of selinexor treatment and require prophylactic supportive care and additional on study management to limit duration and severity. The median onset of N/V occurred at day 3, suggesting delayed emesis, which should be properly managed [28, 29]. Previous studies have suggested that selinexor-induced N/V is likely mediated through the central nervous system, as selinexor crosses the blood brain barrier and has anticancer activity in the brain [30–32]. Prophylactic use of a 5-HT3 RAs (ondansetron or equivalent) in all patients, was mandated on selinexor trials after phase 1 to reduce N/V. The use of a second antiemetic, such as 2.5–10 mg olanzapine in the evening and/or NK1-RAs should be strongly considered prior to the first day of therapy. Additional antinausea medications may be required for patients who experience on study gastrointestinal AEs, and the data shown here suggest that NK1-RAs, benzodiazepines, and cannabinoid-RAs were effective at mitigating treatment-emergent N/V. As suggested by the association data, several AEs are linked to N/V, including decreased appetite, weight loss, hyponatremia, and fatigue, suggesting that management of N/V may limit the incidence of these AEs. Diarrhea was less common in the study population and was generally responsive to loperamide or bismuth subsalicylate.

The constitutional AE of fatigue requires early and vigilant management while patients are receiving selinexor treatment. Physicians should refer to the National Comprehensive Cancer Network (NCCN) Guidelines for the treatment of cancer related fatigue, as well as closely monitor for and correct underlying contributors to fatigue [33]. Methylphenidate at doses of ≥10 mg in the morning proved to be the most effective supportive care strategy for attenuating the symptoms of fatigue, with 8 out of 9 patients never having fatigue or having it resolve with use.

Decreased appetite and weight loss can occur with selinexor treatment and were also the primary side effects observed preclinically in animal models [34]. Nutritional counseling, dose modifications, and appetite stimulants may prevent or improve weight loss. For patients with decreased appetite, megestrol, cannabinoids, and mirtazapine or olanzapine were effective at resolving anorexia and reducing its duration. Physicians should also monitor for corresponding N/V or dysgeusia, which can substantially impact a patient’s desire to eat. Olanzapine (2.5–10 mg) used in the evening and begun prior to the first dose of selinexor appears to address both N/V and decreased appetite.

Hyponatremia is a common AE observed with selinexor treatment with the highest incidence occurring in MM trials. A majority (>95%) of the hyponatremia was asymptomatic, and generally correctable with sodium tablets or electrolyte-containing fluids. The causes of hyponatremia are not completely understood and are likely to be multifactorial. Furthermore, pseudohyponatremia in many cases could not be ruled out and can be caused by elevated paraprotein levels and/or hyperglycemia. Appropriate laboratory methods using direct potentiometry in a gas analyzer are required to correct for pseudohyponatremia.

There are several limitations of this analysis which should be considered when interpreting the results. Firstly, the data were pooled from four independent clinical trials that treated patients with MM at different stages of their disease, and 35% of the patients received combination therapies which could exacerbate AE prevelance and grade. Among these studies, there was a wide range of baseline characteristics, including: prior treatment history (0–3 prior lines; 22%, and ≥4 prior lines, 78% of patients), disease burden, frailty, ECOG performance status, as well as differences in the treatment schedule, dose, and regimen. Previous studies have demonstrated that AE burden increases with each line of therapy, age, and frailty of patients [35]. While the majority of the patients were heavily pretreated (median 6 prior lines of therapy), 22% of the population analyzed received ≤3 prior antimyeloma regimens, which could impact onset, duration, and resolution of specific AEs. An additional limitation to this analysis is that patients may have received multiple interventions, at different times of AE onset, and controlling for these interventions and interpreting the effect in small subsets of patients was challenging. Furthermore, as knowledge of the AE profile of selinexor improved with clinical experience, prophylactic supportive care and early follow-up to mitigate AE severity became more common, resulting in nonstandardized or controlled AE prevention and management.

In conclusion, selinexor is a novel anticancer therapy that is being integrated in the therapeutic algorithm for patients with relapsed/refractory MM. The side effects of selinexor are unique amongst myeloma drugs, and are generally reversible with dose modification and supportive care. Clincial trials are ongoing to investigate selinexor in earlier lines of therapy and at lower doses in combination with approved agents, with the goal of maximizing efficacy and reducing side effects. Further studies examining AE mitigation strategies in a large, standardized patient population will provide further insight and help establish additional guidelines for the management of common AEs related to selinexor treatment.

## Supplementary information

Supplemental Table 1

Supplemental Table 2

Supplemental Figure 1A_D

Supplemental Table 3
