# Supplementary material for: Integrated safety profile of selinexor in multiple myeloma: experience from 437 patients enrolled in clinical trials
Source: Leukemia. 2020 Feb 24;34(9):2430–40. doi: 10.1038/s41375-020-0756-6 (PMC7449872; doi:10.1038/s41375-020-0756-6)
Supplement: Supplementary file 1 — Supplemental Table 1 [file 41375_2020_756_MOESM1_ESM.docx]

**Supplemental Table 1. Inclusion / Exclusion Criteria for Each Trial**

**KCP-330-001 Trial**

**Inclusions:**

1. Written informed consent in accordance with federal, local, and institutional guidelines
2. Age ≥ 18 years
3. Patients with malignancies that are refractory to or who are intolerant of established therapy known to provide clinical benefit for their condition. Patients must not be candidates for anti-tumor regimens known to provide clinical benefit.
4. Histologically confirmed diagnosis, and evidence of disease progression, of multiple myeloma (MM) as described below:

**Multiple Myeloma (MM):** Symptomatic disease previously treated with, and relapsed or refractory to, ≥ 3 prior regimens (lines of therapy) that included at least one of each of the following: an alkylating agent, an immunomodulatory drug, a proteasome inhibitor, and a steroid. Patients must have measurable disease as defined by at least one of the following:

1. Serum M-protein ≥ 0.5 g/dL by serum protein electrophoresis (SPEP) or for IgA myeloma, by quantitative IgA; or
2. Urinary M-protein excretion at least 200 mg/24hours; or
3. Serum Free Light Chain (SFLC) whereby the involved light chain measures ≥ 10 mg/dL and with an abnormal ratio

**Arm 8 Dose Evaluation Cohort:**

Approximately 10 patients with MM in each of the following treatment groups:

Group A – 40mg

Group B – 60mg

Group C – 80mg - Patients with BSA <1.2 should not be enrolled in Group C as their dose will exceed 70mg/m^2^.

Patients must have symptomatic disease previously treated with, and relapsed or refractory to, ≥ 3 prior regimens (lines of therapy) that included at least one of each of the following: an alkylating agent, an immunomodulatory drug, a proteasome inhibitor, and a steroid. Patients who require glucocorticoids for other comorbidities (e.g., asthma) are not eligible for Arm 8. Patients must have measurable disease as defined by at least one of the following:

- Serum M-protein ≥ 0.5 g/dL by serum protein electrophoresis (SPEP) or for IgA myeloma, by quantitative IgA; or
- Urinary M-protein excretion at least 200 mg/24hours; or
- Serum Free Light Chain (SFLC) whereby the involved light chain measures ≥ 10 mg/dL and with an abnormal ratio.

1. All patients on this study must have evidence of progressive disease on study entry. There is no upper limit on the number of prior treatments provided all inclusion/exclusion criteria are met.
2. Eastern Cooperative Oncology Group (ECOG) Performance Status of 0‑1 (Appendix 1).
3. Adequate hepatic function within 14 days prior to C1D1: total bilirubin <2 times the upper limit of normal (ULN) (except patients with Gilbert’s syndrome who must have a total bilirubin of < 3 times ULN), aspartate aminotransferase (AST) < 2.5 times ULN and alanine aminotransferase (ALT) < 2.5 times ULN. In the case of known (radiologically- and/or biopsy-documented) liver metastasis, AST < 5.0 times ULN and ALT < 5.0 times ULN is acceptable.
4. Adequate renal function within 14 days prior to C1D1: estimated creatinine clearance of ≥ 30 mL/min, calculated using the formula of Cockroft and Gault (140-Age) • Mass (kg)/(72 • creatinine mg/dL); multiply by 0.85 if female.
5. Female patients of child-bearing potential must have a negative serum pregnancy test at screening and agree to use reliable methods of contraception for three months after their last dose of medication. Such methods include intrauterine devices, hormonal contraceptives [contraceptive pills, implants, transdermal patches, hormonal vaginal devices or injections with prolonged release], abstinence or sterilization of the partner. Male patients must use a reliable method of contraception (abstinence or contraception with one of the above-described methods for your partner), if sexually active with a female of child-bearing potential. Total (true) abstinence (when this is in line with the preferred and usual lifestyle of the patient), is an acceptable method of contraception. Periodic abstinence (e.g., calendar, ovulation, symptothermal, post-ovulation methods) and withdrawal are not acceptable methods of contraception. For both male and female patients, effective methods of contraception must be used throughout the study and for three months following the last dose.
6. Patients receiving hematopoietic growth factor support including erythropoietin, darbepoetin, G-CSF, GM-CSF, and platelet stimulators can continue to do so, but must be transfusion independent for at least 3 weeks prior to registration in the dose escalation cohorts of the study. Elective transfusions are permitted for transfusion independent patients during the 3-week period prior to dosing, except in the dose escalation cohorts. There are no restrictions on transfusions for enrollment into and during the dose expansion and dose evaluation cohorts of the study. Screening absolute neutrophil count (ANC) should be independent of growth factor support for at least 1 week prior to registration for all patients.
7. Adequate hematopoietic function within 14 days prior to C1D1: total white blood cell (WBC) count ≥ 1,500/mm^3^, absolute neutrophil count (ANC) ≥ 800/mm^3^, hemoglobin (Hb) ≥ 8.0gm/dL, and platelet count ≥ 30,000/mm^3^. Screening ANC should be independent of growth factor support for > 1 week for all patients.

**Exclusions:**

Patients meeting any of the following exclusion criteria are not eligible to enroll in this study.

1. Patients who are pregnant or lactating;
2. Radiation, chemotherapy, or immunotherapy or any other anticancer therapy ≤ 2 weeks prior to cycle 1 day 1 and mitomycin C and radio-immunotherapy 6 weeks prior to cycle 1 day 1.
3. Patients with active graft versus host disease after allogeneic stem cell transplantation. At least 3 months must have elapsed since completion of allogeneic stem cell transplantation
4. Major surgery within four weeks before Day 1
5. Unstable cardiovascular function:

- symptomatic ischemia, or
- uncontrolled clinically significant conduction abnormalities (i.e., ventricular tachycardia on antiarrhythmics are excluded and 1st degree AV block or asymptomatic LAFB/RBBB will not be excluded), or
- congestive heart failure (CHF) of NYHA Class ≥ 3, or
- myocardial infarction (MI) within 3 months;

1. Uncontrolled active infection requiring parenteral antibiotics, antivirals, or antifungals within one week prior to first dose;
2. Known to be HIV seropositive;
3. Known active hepatitis A, B, or C infection; or known to be positive for HCV RNA or HBsAg (HBV surface antigen);
4. Patients with active CNS malignancy. Asymptomatic small lesions are not considered active. Treated lesions may be considered inactive if they are stable for at least 3 months. Patients with malignant cells in their cerebrospinal fluid (CSF) without CNS symptoms may be included.
5. Patients with significantly diseased or obstructed gastrointestinal tract or uncontrolled vomiting or diarrhea.
6. Grade ≥ 2 peripheral neuropathy at baseline (within 14 days prior to cycle 1 day 1).
7. History of seizures, movement disorders or cerebrovascular accident within the past 5 years prior to cycle 1 day 1.
8. Patients with macular degeneration, uncontrolled glaucoma, or markedly decreased visual acuity
9. In the opinion of the Investigator, patients who are significantly below their ideal body weight.
10. Serious psychiatric or medical conditions that could interfere with treatment;
11. Treatment with an investigational anti-cancer drug within 3 weeks prior to first dose of study drug;
12. Concurrent therapy with approved or investigational anticancer therapeutic other than steroids or hydroxyurea as specified above in exclusion #2.
13. For patients screening for Arm 8 **Group C** (80mg) only: BSA <1.2

**KCP-330-012**

**Inclusion/Exclusion Criteria:**

*Inclusion:*

Patients must meet all of the following inclusion criteria to be eligible to enroll in this study:

1. Written informed consent in accordance with federal, local, and institutional guidelines.
2. Age ≥ 18 years at the time of signing informed consent.
3. Measurable MM based on IMWG guidelines as defined by at least one of the following:

a. Serum M-protein ≥ 0.5 g/dL by serum electrophoresis (SPEP) or, for IgA myeloma, by quantitative IgA

b. Urinary M-protein excretion ≥ 200 mg/24 hours

c. FLC ≥ 100 mg/L, provided that the FLC ratio is abnormal.

d. If serum protein electrophoresis is felt to be unreliable for routine M-protein measurement, then quantitative Ig levels by nephelometry is acceptable.

1. Patients must have previously received ≥ 3 anti-MM regimens including: an alkylating agent, lenalidomide, pomalidomide, bortezomib, carfilzomib, daratumumab, and a glucocorticoid. There is no upper limit on the number of prior therapies provided that all other inclusion/exclusion criteria are met.
2. MM refractory to previous treatment with one or more glucocorticoids, parenteral PI (i.e., bortezomib and/or carfilzomib), IMiD (i.e., lenalidomide and/or pomalidomide), and daratumumab (except quad-refractory in Part A). Refractory is defined as ≤ 25% response to therapy, or progression during therapy or progression within 60 days after completion of therapy.
3. Multiple myeloma that is refractory to the patient’s most recent anti-MM regimen. (Documented severe intolerance to the patient’s last therapy is allowed upon approval by the Medical Monitor.)
4. Any clinically significant non-hematological toxicities (except for peripheral neuropathy as described in exclusion criterion #17) that patients experienced from treatments in previous clinical studies must have resolved to Grade ≤ 2 by Cycle 1 Day 1.
5. Adequate hepatic function within 21 days prior to Cycle 1 Day 1: total bilirubin < 2x upper limit of normal (ULN) (except patients with Gilbert’s syndrome who must have a total bilirubin of < 3x ULN), AST < 2.5x ULN and ALT < 2.5x ULN.
6. Adequate renal function within 21 days prior to Cycle 1 Day 1: estimated creatinine clearance of ≥ 20 mL/min, calculated using the formula of Cockroft and Gault.
7. Female patients of childbearing potential must agree to use 2 methods of contraception (including 1 highly effective and 1 effective method of contraception) and have a negative serum pregnancy test at Screening. Male patients must use an effective barrier method of contraception if sexually active with a female of child-bearing potential. For both male and female patients, effective methods of contraception must be used throughout the study and for three months following the last dose of study treatment.
8. Eastern Cooperative Oncology Group (ECOG) Performance Status of ≤ 2.
9. Adequate hematopoietic function within 21 days prior to Cycle 1 Day 1 (See Exclusion Criterion #20 for transfusion washout periods for RBCs and platelets):
   a. Total WBC count > 1,000/mm^3^

b. ANC ≥ 1000/mm^3^

c. Platelet count ≥ 75,000/mm^3^ (patients in whom < 50% of bone marrow nucleated cells are plasma cells) or ≥ 50,000/mm^3^ (patients in whom ≥ 50% of bone marrow nucleated cells are plasma cells. [Platelet transfusions < 1 week prior to Cycle 1 Day 1 are prohibited (see below).]

1. Hemoglobin level ≥ 8.5 g/dL. In certain cases, patients with stable baseline hemoglobin level > 8.0 may be included following approval by the Medical Monitor. [Red blood cell transfusions < 2 weeks prior to Cycle 1 Day 1 are prohibited (see below).]
2. Confirmation of patient eligibility for specific key criteria for study participation with the Medical Monitor.

*Exclusion Criteria:*

1. Active smoldering MM.
2. Active plasma cell leukemia.
3. Documented systemic amyloid light chain amyloidosis.
4. Active central nervous system (CNS) MM.
5. Pregnancy or breastfeeding.
6. Radiation, chemotherapy, or immunotherapy or any other anticancer therapy ≤ 2 weeks prior to Cycle 1 Day 1, and radio-immunotherapy 6 weeks prior to Cycle 1 Day 1.
7. Active graft vs. host disease (after allogeneic stem cell transplantation) at Cycle 1 Day 1
8. Life expectancy of < 4 months.
9. Major surgery within four weeks prior to Cycle 1 Day 1.
10. Active, unstable cardiovascular function:

a. Symptomatic ischemia, or

b. Uncontrolled clinically-significant conduction abnormalities (e.g., patients with ventricular tachycardia on antiarrhythmics are excluded; patients with 1st degree atrioventricular (AV) block or asymptomatic left anterior fascicular block/right bundle branch block (LAFB/RBBB) will not be excluded), or

c. Congestive heart failure (CHF) of New York Heart Association (NYHA) Class ≥ 3, or

d. Myocardial infarction (MI) within 3 months prior to Cycle 1 Day 1.

1. Active, uncontrolled hypertension.
2. Uncontrolled active infection requiring parenteral antibiotics, antivirals, or antifungals within one week prior to first dose.
3. Known HIV seropositive.
4. Known active hepatitis A, B, or C infection; or known to be positive for HCV RNA or HBsAg (HBV surface antigen).
5. Prior malignancy that required treatment, or has shown evidence of recurrence (except for non-melanoma skin cancer or adequately treated cervical carcinoma in situ) during the 5 years prior to enrollment. Cancer treated with curative intent > 5 years previously and without evidence of recurrence will be allowed.
6. Active GI dysfunction interfering with the ability to swallow tablets, or any GI dysfunction that could interfere with absorption of study treatment.
7. Grade ≥ 3 peripheral neuropathy, and Grade ≥ 2 painful neuropathy, within 21 days prior to Cycle 1 Day 1.
8. Serious, active psychiatric or medical conditions which, in the opinion of the Investigator, could interfere with treatment.
9. Participation in an investigational anti-cancer study within 21 days prior to Cycle 1 Day 1.
10. Receipt of transfusions as follows:
11. Platelet infusion within 1 week prior to Cycle 1 Day 1.
12. RBC transfusion within 2 weeks prior to Cycle 1 Day 1.
13. Receipt of the following blood growth factors within 2 weeks prior to Cycle 1 Day 1: Granulocyte colony stimulating factor (G-CSF), granulocyte-macrophage colony stimulating factor (GM-CSF), erythropoietin (EPO), or megakaryocyte growth factor.
14. Known intolerance to or contraindication for glucocorticoid therapy at Cycle 1 Day 1.
15. Prior exposure to a SINE compound, including selinexor.
16. Unable or unwilling to comply with protocol requirements, including providing a 24‑hour urine samples at the required study time points.

*Documentation Requirements*

For enrollment consideration, patients may be eligible if they have *documented* *evidence* of previous treatment for MM that substantiates disease status as follows:

- Refractory to a glucocorticoid
- Refractory to lenalidomide and/or pomalidomide (only 1 required) *and* evidence of prior treatment* with *both* lenalidomide and pomalidomide
- Refractory to bortezomib and/or carfilzomib (only 1 required) *and* evidence of prior treatment* with *both* bortezomib and carfilzomib
- Refractory to daratumumab (except for quad-refactory patients in Part A)

* Prior treatment is defined as either refractory, treatment with ≥ 2 cycles, or documented severe intolerance.

*Documented evidence will include at least one of the following:*

1. Medical records that support start and stop dates (month/year) of prior treatment (both dose and schedule), best response on prior treatment and, if applicable, date of progression (including both dose and schedule at the time of progression).
2. Myeloma marker values (SPEP, UPEP, Immunoglobulin, FLC) at the time of prior treatment start, stop and time of progression (accompanied by #1).
3. Formal, signed physician letter by Investigator (on hospital/clinic letterhead), to be included in the patient’s medical and research record, indicating start/stop dates of prior treatment (both dose and schedule), best response on treatment and, if applicable, date of progression (including both dose and schedule at the time of progression).
4. Formal, signed physician letter from referring physician (on hospital/clinic letterhead), to be included in the patient’s medical and research record, that includes prior treatment history indicating start/stop dates of prior treatment (both dose and schedule), best response on prior treatment and, if applicable, date of progression (including both dose and schedule at the time of progression).

**KCP-330-017**

**Inclusion/Exclusion Criteria**

***Inclusion***

1. Written informed consent in accordance with federal, local, and institutional guidelines
2. Age ≥ 18 years at the time of informed consent
3. Histologically confirmed diagnosis, measurable disease and evidence of disease progression of MM, as described below:
4. Symptomatic MM, based on IMWG guidelines. Patients must have measurable disease as defined by at least one of the following:
5. Serum M-protein ≥ 0.5 g/dL by serum protein electrophoresis (SPEP) or, for IgA myeloma, by quantitative IgA
6. Urinary M-protein excretion at least 200 mg/24 hours
7. Serum FLC ≥ 100 mg/L, provided that FLC ratio is abnormal
8. If SPEP is felt to be unreliable for routine M-protein measurement (e.g., for IgA MM), then quantitative Ig levels by nephelometry or turbidometry are acceptable
9. Any non-hematological toxicities (except for peripheral neuropathy as described in exclusion criterion #24) that patients had from treatments in previous clinical studies must have resolved to ≤ Grade 2 by C1D1.
10. Eastern Cooperative Oncology Group (ECOG) Performance Status of ≤ 2
11. Adequate hepatic function within 21 days prior to C1D1:

- For SPd and SRd: Total bilirubin < 2x upper limit of normal (ULN) (except patients with Gilbert’s syndrome [hereditary indirect hyperbilirubinemia] who must have a total bilirubin of ≤ 3x ULN) and both aspartate aminotransferase (AST) and alanine aminotransferase (ALT) < 2.5x ULN
- For SVd and SDd: Total bilirubin < 1.5x ULN (except patients with Gilbert’s syndrome [hereditary indirect hyperbilirubinemia] who must have a total bilirubin of ≤ 3x ULN) and both AST and ALT < 2.0x ULN

1. Adequate renal function within 21 days prior to C1D1:

- Estimated creatinine clearance of (calculated using the formula of Cockroft and Gault (*Cockroft and Gault 1976*):
- ≥ 20 mL/min for SVd, SPVd, SDd, and SKd Arms
- ≥ 45 mL/min for SPd Arm (as requested by the manufacturer)
- > 50 mL/min for SRd Arm

1. Adequate hematopoietic function within 21 days prior to C1D1: total white blood cell (WBC) count ≥ 1,500/mm^3^, ANC ≥ 1000/mm^3^, hemoglobin (Hb) ≥ 8.0 g/dL, and platelet count ≥ 75,000/mm^3^. For expansion cohorts only, platelet counts > 50,000/mm^3^; for patients in whom ≥ 50% of bone marrow nucleated cells are plasma cells, platelets ≥ 30,000/mm^3^ are acceptable for expansion cohorts. Patients receiving hematopoietic growth factor support, including erythropoietin (EPO), darbepoetin, granulocyte-colony stimulating factor (G-CSF), granulocyte macrophage-colony stimulating factor (GM-CSF), and platelet stimulators (e.g., eltrombopag or romiplostim) may continue to do so. However, patients in the escalation cohorts must be platelet transfusion independent for > 1 week in order to be enrolled in the study.
2. Female patients of childbearing potential must have a negative serum pregnancy test at Screening Female patients of childbearing potential and fertile male patients who are sexually active with a female of childbearing potential must use highly effective methods of contraception throughout the study and for 3 months following the last dose of study treatment.

*SPd (Arm 1) Only:*

1. Relapsed and refractory MM with:
2. Documented evidence of PD after achieving at least stable disease (SD) for ≥ 1 cycle during a previous MM regimen (i.e., relapsed MM)
3. ≤ 25% response (i.e., patients never achieved ≥ MR) or PD during or within 60 days from the end of the most recent MM regimen (i.e., refractory MM)
4. Previously undergone ≥ 2 cycles of lenalidomide and a PI (in separate therapeutic regimens [not for maintenance] or in combination)
5. In the expansion arm at RP2D, patients must not be pomalidomide refractory

*SVd (Arm 2) Only:*

1. Relapsed or refractory MM with:
2. Documented evidence of relapse after ≥ 1 previous line of therapy
3. Not refractory to bortezomib in their most recent line of therapy

*SRd in RRMM (Arm 3) Only:*

1. Patients who received ≥ 1 prior therapeutic regimen (prior lenalidomide is allowed as long as patient’s MM was not refractory to prior lenalidomide; patients whose MM was refractory to lenalidomide maintenance regimens will be allowed in this cohort).

*SDd (Arm 5) Only:*

1. Patients who received ≥ 3 prior lines of therapy, including a PI and an IMiD, or patients with MM refractory to both a PI and an IMiD.
2. Patients must not have received prior anti-CD38 monoclonal antibodies (Cohort 5.3 ONLY – Dose Expansion at RP2D).

**Exclusion:**

- 1. Smoldering MM
  2. MM that does not express M-protein or FLC (i.e., non-secretory MM is excluded), and quantitative immunoglobulin levels cannot be used instead
  3. Documented active systemic amyloid light chain amyloidosis
  4. Active plasma cell leukemia
  5. Blood (or blood product) transfusions and blood growth factors within 7 days of C1D1 (only for patients enrolling into the Expansion Phase)
  6. Radiation, chemotherapy, or immunotherapy or any other anticancer therapy ≤ 2 weeks prior to C1D1, and radio-immunotherapy within 6 weeks prior to C1D1. Patients on long-term glucocorticoids during Screening do not require a washout period. Prior radiation is permitted for treatment of fractures or to prevent fractures as well as for pain management
  7. Patients with history of SCC with residual paraplegia (Dose Escalation Phase only)
  8. Treatment with an investigational anti-cancer therapy within 3 weeks prior to C1D1
  9. Prior autologous stem cell transplantation < 1 month, or allogeneic stem cell transplantation < 3 months prior to C1D1
  10. Active graft versus host disease after allogeneic stem cell transplantation
  11. Life expectancy < 3 months
  12. Major surgery within 4 weeks prior to C1D1
  13. Active, unstable cardiovascular function:

1. Symptomatic ischemia, or
2. Uncontrolled, clinically significant conduction abnormalities (e.g., patients with ventricular tachycardia on antiarrhythmics are excluded; patients with 1st degree atrioventricular (AV) block or asymptomatic left anterior fascicular block/right bundle branch block (LAFB/RBBB) will not be excluded), or
3. Congestive heart failure (CHF) of New York Heart Association (NYHA) Class ≥ 3, or
4. Myocardial infarction (MI) within 3 months prior to C1D1, or
5. Ejection fraction (EF) < 50% at Screening
   1. Uncontrolled active hypertension
   2. Uncontrolled active infection requiring parenteral antibiotics, antivirals, or antifungals within 1 week prior to first dose
   3. Known active hepatitis A, B, or C
   4. Known human immunodeficiency virus (HIV) infection or HIV seropositivity
   5. Any active gastrointestinal dysfunction that prevents the patient from swallowing tablets, or interferes with absorption of study treatment
   6. Currently pregnant or breastfeeding
   7. A serious psychiatric or medical condition which, in the opinion of the Investigator, could interfere with treatment
   8. Hypersensitivity to any of the treatments for the Arm in which the patient is enrolled
   9. *In the SVd Arm*: Prior history of neuropathy Grade > 2, or Grade ≥ 2 neuropathy with pain at Screening (within 21 days prior to C1D1)
   10. Prior exposure to a SINE compound, including selinexor

**KCP-330-023**

- 1. Inclusion Criteria

Patients must meet all of the following inclusion criteria to be eligible to enroll in this study:

1. Histologically confirmed MM with measurable disease per IMWG guidelines as defined by at least 1 of the following:
   1. Serum M-protein ≥ 0.5 g/dL (> 5 g/L) by serum protein electrophoresis (SPEP) or for immunoglobulin (Ig) A myeloma, by quantitative serum IgA levels; or
   2. Urinary M-protein excretion at least 200 mg/24 hours; or
   3. Serum FLC ≥ 100 mg/L, provided that the serum FLC ratio is abnormal (normal FLC ratio: 0.26 to 1.65).
2. Had at least 1 prior anti-MM regimen and no more than 3 prior anti-MM regimens. Induction therapy followed by stem cell transplant and consolidation/maintenance therapy will be considered as 1 anti-MM regimen.
3. Documented evidence of progressive MM (based on the Investigator's determination according to the IMWG response criteria) on or after their most recent regimen.
4. Prior treatment with bortezomib or other PI is allowed, provided all of the following criteria are met:
   - Best response achieved with prior bortezomib at any time was ≥ PR and with the last PI therapy (alone or in combination) was ≥ PR, AND
   - Participant did not discontinue bortezomib due to Grade ≥ 3 related toxicity, AND
   - Must have had at least a 6‑month PI-treatment-free interval prior to C1D1 of study treatment.
5. Must have an ECOG Status score of 0, 1, or 2.
6. Written informed consent in accordance with federal, local, and institutional guidelines.
7. Age ≥ 18 years.
8. Resolution of any clinically significant non-hematological toxicities (if any) from previous treatments to Grade ≤ 1 by C1D1. Patients with chronic, stable Grade 2 non‑hematological toxicities may be included following approval from the Medical Monitor.
9. Adequate hepatic function within 28 days prior to C1D1:
   1. Total bilirubin < 1.5 × upper limit of normal (ULN) (except patients with Gilbert’s syndrome who must have a total bilirubin of < 3 × ULN), and
   2. Aspartate aminotransferase (AST) and alanine aminotransferase (ALT) normal to < 2 × ULN.
10. Adequate renal function within 28 days prior to C1D1 (estimated creatinine clearance [CrCl] of ≥ 20 mL/min, calculated using the formula of Cockroft and Gault):

(140-Age) × Mass (kg)/(72 × creatinine mg/dL)

Multiply by 0.85 if the patient is female, or if CrCl is ≥ 20 mL/min as measured by 24‑hour urine collection.

1. Adequate hematopoietic function within 7 days prior to C1D1: total white blood cell (WBC) count ≥ 1500/mm^3^, absolute neutrophil count ≥ 1000/mm^3^, hemoglobin ≥ 8.5 g/dL and platelet count ≥ 75,000/mm^3^ (patients for whom < 50% of bone marrow nucleated cells are plasma cells) or ≥ 50,000/mm^3^ (patients for whom ≥ 50% of bone marrow nucleated cells are plasma cells).
   1. Patients receiving hematopoietic growth factor support, including erythropoietin, darbepoetin, granulocyte-colony stimulating factor (G-CSF), granulocyte macrophage-colony stimulating factor (GM-CSF), and platelet stimulators (e.g., eltrombopag, romiplostim, or interleukin-11) must have a 2-week interval between growth factor support and the Screening assessments, but they may receive growth factor support during the study.
   2. Patients must have:
   - At least a 2-week interval from the last red blood cell (RBC) transfusion prior to the Screening hemoglobin assessment, and
   - At least a 1-week interval from the last platelet transfusion prior to the Screening platelet assessment.

However, patients may receive RBC and/or platelet transfusions as clinically indicated per institutional guidelines during the study.

1. Female patients of childbearing potential must agree to use 2 methods of contraception (including 1 highly effective and 1 effective method of contraception) and have a negative serum pregnancy test at Screening. Male patients must use an effective barrier method of contraception if sexually active with a female of childbearing potential. For both male and female patients, effective methods of contraception must be used throughout the study and for 3 months following the last dose of study treatment.
   1. Exclusion Criteria

Patients meeting any of the following exclusion criteria are not eligible to enroll in this study:

1. Prior exposure to a SINE compound, including selinexor.
2. Prior malignancy that required treatment, or has shown evidence of recurrence (except for non-melanoma skin cancer or adequately treated cervical carcinoma in situ) during the 5 years prior to randomization. Cancer treated with curative intent for > 5 years previously and without evidence of recurrence will be allowed.
3. Has any concurrent medical condition or disease (e.g., uncontrolled active hypertension, uncontrolled active diabetes, active systemic infection, etc.) that is likely to interfere with study procedures.
4. Uncontrolled active infection requiring parenteral antibiotics, antivirals, or antifungals within 1 week prior to C1D1. Patients on prophylactic antibiotics or with a controlled infection within 1 week prior to C1D1 are acceptable.
5. Active plasma cell leukemia.
6. Documented systemic light chain amyloidosis.
7. MM involving the central nervous system.
8. Polyneuropathy, organomegaly, endocrinopathy, monoclonal gammopathy, and skin changes (POEMS) syndrome.
9. Spinal cord compression.
10. Greater than Grade 2 peripheral neuropathy or Grade ≥ 2 peripheral neuropathy with pain at baseline, regardless of whether or not the patient is currently receiving medication.
11. Known intolerance, hypersensitivity, or contraindication to glucocorticoids.
12. Radiation, chemotherapy, or immunotherapy or any other anticancer therapy (including investigational therapies) ≤ 2 weeks prior to C1D1. Localized radiation to a single site at least 1 week before C1D1 is permitted. Glucocorticoids within 2 weeks of C1D1 are permitted. Patients on long-term glucocorticoids during Screening do not require a washout period but must be able to tolerate the specified dexamethasone dose in this study.
13. Prior autologous stem cell transplantation < 1 month or allogeneic stem cell transplantation < 4 months prior to C1D1.
14. Active graft versus host disease (after allogeneic stem cell transplantation) at C1D1.
15. Pregnant or breastfeeding females.
16. BSA < 1.4 m^2^ at baseline, calculated by the Dubois ([Dubois and Dubois, 1916](#_ENREF_9)) or Mosteller ([Mosteller, 1987](#_ENREF_21)) method.
17. Life expectancy of < 4 months.
18. Major surgery within 4 weeks prior to C1D1.
19. Active, unstable cardiovascular function:
    1. Symptomatic ischemia, or
    2. Uncontrolled clinically significant conduction abnormalities (e.g., patients with ventricular tachycardia on anti-arrhythmics are excluded; patients with first‑degree atrioventricular block or asymptomatic left anterior fascicular block/right bundle branch block will not be excluded), or
    3. Congestive heart failure of New York Heart Association Class ≥ 3 or known left ventricular ejection fraction < 40%, or
    4. Myocardial infarction within 3 months prior to C1D1.
20. Known active human immunodeficiency virus (HIV) infection or HIV seropositivity.
21. Known active hepatitis A, B, or C infection; or known to be positive for hepatitis C virus ribonucleic acid (RNA) or hepatitis B virus surface antigen.
22. Any active gastrointestinal dysfunction interfering with the patient’s ability to swallow tablets, or any active gastrointestinal dysfunction that could interfere with absorption of study treatment.
23. Any active, serious psychiatric, medical, or other conditions/situations that, in the opinion of the Investigator, could interfere with treatment, compliance, or the ability to give informed consent.
24. Contraindication to any of the required concomitant drugs or supportive treatments.
25. Patients unwilling or unable to comply with the protocol, including providing 24-hour urine samples for urine protein electrophoresis at the required time points.
