# Supplementary material for: Integrated safety profile of selinexor in multiple myeloma: experience from 437 patients enrolled in clinical trials
Source: Leukemia. 2020 Feb 24;34(9):2430–40. doi: 10.1038/s41375-020-0756-6 (PMC7449872; doi:10.1038/s41375-020-0756-6)
Supplement: Supplementary file 2 — Supplemental Table 2 [file 41375_2020_756_MOESM2_ESM.docx]

**Supplemental Table 2. Adverse Events by Treatment Regimen**
