# Supplementary material for: Integrated safety profile of selinexor in multiple myeloma: experience from 437 patients enrolled in clinical trials
Source: Leukemia. 2020 Feb 24;34(9):2430–40. doi: 10.1038/s41375-020-0756-6 (PMC7449872; doi:10.1038/s41375-020-0756-6)
Supplement: Supplementary file 3 — Supplemental Figure 1A_D [file 41375_2020_756_MOESM3_ESM.docx]

**Supplemental Figure 1. Effect of Intervention on Platelet Counts**

**A.** **Platelet Transfusion**

**
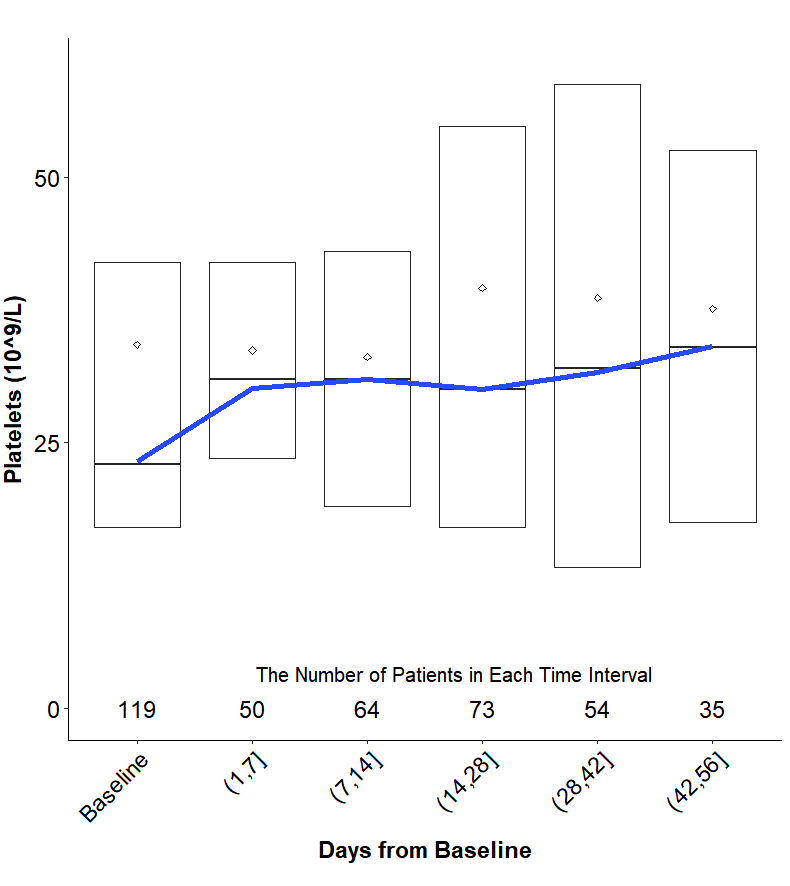
**

**B. Thrombopoietin (TPO) Receptor Agonist**

**
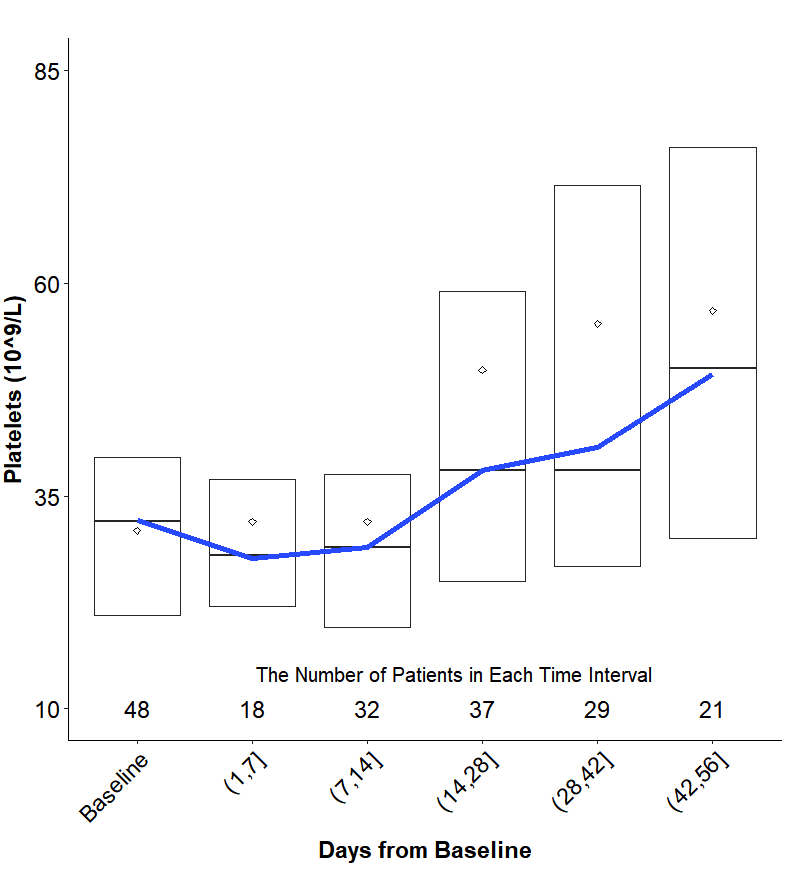
**

**C. Dose Reduction

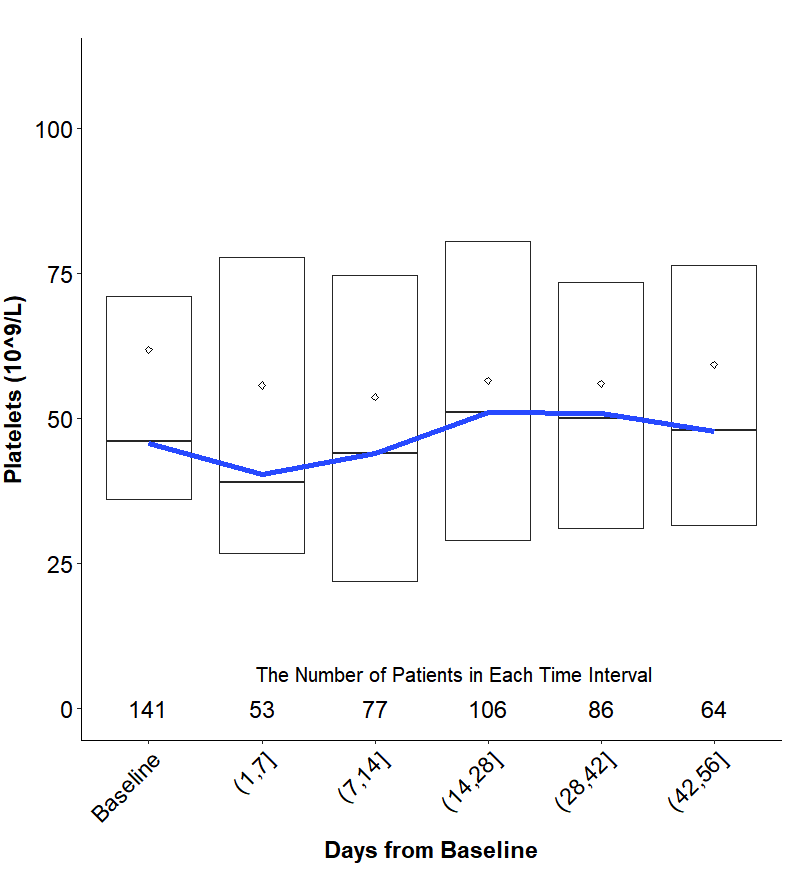
**

**D. Dose Interruption**

**
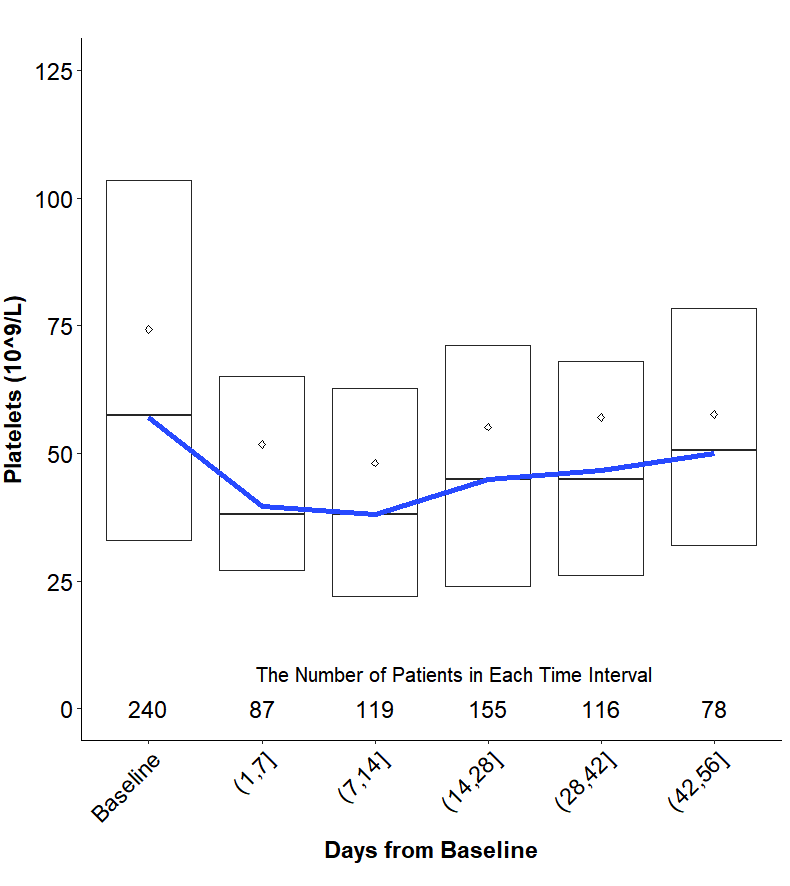
**

**Figure 1 A, B, C, D**. Change in platelet levels after a (A) platelet transfusion, (B) TPO receptor agonist, (C) dose reduction, or (D) dose interruption. Baseline is the time point at which the intervention began while on study.
