# Supplementary material for: Integrated safety profile of selinexor in multiple myeloma: experience from 437 patients enrolled in clinical trials
Source: Leukemia. 2020 Feb 24;34(9):2430–40. doi: 10.1038/s41375-020-0756-6 (PMC7449872; doi:10.1038/s41375-020-0756-6)
Supplement: Supplementary file 4 — Supplemental Table 3 [file 41375_2020_756_MOESM4_ESM.docx]

**Supplemental Table 3. Outcomes of Patients Who Received Methylphenidate for Fatigue**

Outcomes of patients who received methylphenidate as a prophylactic or on-study supportive care agent for fatigue. N/A, not applicable. – indicates that fatigue was not resolved or resolving while patient was on-treatment or for the 30 days of post-treatment follow-up.
